# Barriers and facilitators to implementing veterinary telemedicine in animal production

**DOI:** 10.3389/fvets.2024.1452653

**Published:** 2024-11-20

**Authors:** Ana Guintard, Sébastien Assié, Lucile Lefèvre, Maxime Delsart, Benoit Dile, Nikky Millar

**Affiliations:** ^1^Oniris, Centre Hospitalier Universitaire Vétérinaire (CHUV), Nantes, France; ^2^Chambre d’agriculture des Pays de la Loire, Segré-en-Anjou-Bleu, France; ^3^EPIMIM, Laboratoire de Santé Animale, Anses, Ecole Nationale Vétérinaire d’Alfort, Maisons-Alfort, France; ^4^Labovet Conseil, Beaupréau-en-mauges, France; ^5^Department of Pathology and Microbiology, Faculty of Veterinary Medicine, Université de Montréal, Saint-Hyacinthe, QC, Canada; ^6^Groupe de recherche en épidémiologie des zoonoses et santé publique (GREZOSP), Faculty of Veterinary Medicine, Université de Montréal, Saint-Hyacinthe, QC, Canada; ^7^Centre de recherche en santé publique, Université de Montréal et Centre intégré de santé et de services sociaux du Québec du Centre-Sud-de-l’Île-de-Montréal, Montréal, QC, Canada; ^8^Regroupement FRQNT Op+lait, Saint-Hyacinthe, QC, Canada

**Keywords:** veterinary telemedicine, livestock, behavior change, COM-B model, qualitative research teleconsultation, telesurveillance, tele-expertise, teleassistance

## Abstract

In France, in recent years, the field of veterinary medicine has witnessed a growing interest in veterinary telemedicine, driven by rapid technological advancements and a decline in the availability of veterinarians, particularly in remote and rural areas. However, there is a scarcity of literature addressing the barriers and facilitators of implementing telemedicine in veterinary practice. Therefore, this study aims to investigate the factors that influence the adoption of veterinary telemedicine for bovine, poultry and swine in France. Insights from both farmers and veterinarians were collected and subjected to qualitative analysis utilizing the COM-B model of behavior change. Significant barriers and facilitators were identified. Major hurdles encompass technological limitations, regulatory complexities, and concerns regarding the evolution of the veterinary profession. Conversely, the expertise of veterinarians, coupled with their understanding of their clients’ farms, and the potential for remote interventions, emerged as primary facilitators. The study emphasizes the critical role of regulation in ensuring ethical standards and maximizing the benefits of telemedicine. With clear regulatory frameworks in place, telemedicine holds promise for enhancing animal health and optimizing veterinary practice.

## 1 Introduction

Telemedicine, as defined by the World Health Organization (WHO) in 1998, encompasses the use of digital communication technologies for remote medical consultations and procedures, constituting a fundamental component of contemporary healthcare. While its origins can be traced back to antiquity, with historical instances of medical communication through methods like smoke signals, the evolution of technologies such as the telephone and telegraph facilitated the development of modern telemedicine, enabling medical assistance to be provided over long distances (1). In human healthcare, telemedicine plays an important role in extending medical services to remote areas where access to healthcare is limited, including islands, rural regions, and prisons (2). In France, telemedicine has been subject to legal regulation since 2009, with a decree (3) outlining the permissible acts within the realm of telemedicine and defining five distinct branches: teleconsultation, telesurveillance, tele-expertise, teleassistance, and tele-regulation.

In parallel with the growing interest in human telemedicine, veterinary telemedicine is also gaining progress, even though its current status in France remains prohibited. The onset of the COVID-19 pandemic has catalyzed an increased interest in this field, prompting a temporary experimentation phase authorized by decree between May 2020 and November 2021 (4). This decree enabled veterinarians to engage in telemedicine practices through a formal declaration process, facilitating teleconsultations and telesurveillance. As of November 2021, the decree governing the experimental phase of veterinary telemedicine has lapsed, leaving no extant legislation governing veterinary telemedicine practices (5). Nevertheless, a post-experimentation report advocated for the continued utilization of veterinary telemedicine (6).

In the face of regulatory ambiguity surrounding veterinary telemedicine, our study efforts to elucidate the factors influencing its adoption. This study targeted the livestock farming sector, which has witnessed limited integration of veterinary telemedicine, despite its potential to bolster veterinary services and enhance animal health and welfare outcomes through the utilization of livestock sensor data. Thus, the aim of our investigation is to discern the primary facilitators and barriers to the implementation of veterinary telemedicine by gathering the perspectives of bovine, swine, and poultry farmers and veterinarians. The swine and poultry will be refer as monogastric through this article.

## 2 Materials and methods

### 2.1 Researchers contributions and backgrounds

The researchers involved in this study included two agronomists (A.G., L.L.) and four veterinarians (S.A., M.D., B.D., N.M.). One of the authors, S.A., is specialized in bovine medicine and epidemiology. M.D. is specialized in swine medicine, B.D. is specialized in poultry medicine, and one veterinarian, N.M., holds advanced degree in epidemiology and qualitative research. The first author (A.G.) performed the focus groups, data analysis and manuscript writing. A.G. is a woman, with a master in animal production. She attended a training on focus groups before conducting the study.^1^ A.G. had no prior acquaintance with any of the interviewed veterinarians or farmers.

### 2.2 Study design and theoretical framework

A qualitative case study design using focus groups with monogastric and bovine farmers and veterinarians was conducted. The study aimed to investigate barriers and facilitators to the implementation of veterinary medicine for livestock based on the COM-B model. The COM-B model of behavior change provides a theoretical framework to characterize, design and analyze behavior change interventions. It suggests that behavior is influenced by an individual’s capabilities (psychological and physical), opportunities (physical and social) and motivations (automatic and reflective) (7).

In the context of this study, physical capability referred to the physical skills and ability that either limits or enhances the adoption of veterinary telemedicine practices. Psychological capability refers to the knowledge of farmers and veterinarian to implement veterinary telemedicine. Physical opportunity is defined as the external circumstances that affect the adoption of veterinary telemedicine practices. Social opportunity encompasses all the social factors that influence the implementation of telemedicine. Automatic motivation refers to automatic factors, such as feeling, influencing the adoption of veterinary telemedicine practices. Reflective motivation is defined as any reflective process that enhances or limits the adoption of veterinary telemedicine practices.

The study protocol was not subjected to review by the Oniris’ ethical board (Comité d’éthique et de recherche vétérinaire d’Oniris). The board deemed the review unnecessary given the nature of the study, which did not involve animals, and opted not to assess the subject matter.

### 2.3 Participants’ sampling

In this study, the aim was to recruit eight participants per focus group, as it is commonly found in the literature (8). The minimum number of participants was set at four. Both veterinarians and farmers were recruited by researchers from areas close enough for them to attend the half-day sessions in Angers or Segré (France). In addition to geographical criteria, an equal sex ratio was targeted. Initial contact with farmers and veterinarians primarily involved those who were already participating in other projects affiliated with either the veterinary school or the chamber of agriculture. A convenience sampling with snowball effect approach was then used to contact both farmers and veterinarians. Invitations to participate were sent via email to veterinarians, while farmers were contacted by phone.

In addition to participants, one telemedicine expert of each species was invited to attend the focus group to provide information about regulations and address participants’ questions regarding standards and norms surrounding telemedicine in France. bovine and swine experts were teachers at the veterinary school of Nantes and Alfort while the poultry expert was a practitioner veterinarian and a representative of the “Ordre des vétérinaires” (veterinary council), which is, in France, a regulated professional organization similar to the medical or bar associations for physicians and lawyers, respectively. Its primary function is to ensure that its members adhere to professional and ethical standards, thus maintaining the quality of veterinary care. Membership in the Order is mandatory for all practicing veterinarians in France.

Oral consent was obtained and recorded from each participant at the beginning of the focus group. Additionally, the focus groups were entirely recorded.

### 2.4 Data collection

To gather participants’ opinion, data collection involved focus groups discussion surrounding the implementation of telemedicine in the veterinary field. We conducted homogeneous groups because we aimed to foster an environment where participants could share their insights freely, thereby enhancing the quality of the data collected and ensuring that the discussions remained relevant and focused (9). Participants in each focus group were similar regarding their profession (veterinarian or farmer) and species specialization (bovine or monogastric). Four different focus groups were implemented: one group of bovine veterinarians, one group of bovine farmers, one group of monogastric (poultry and swine) veterinarians and one group of monogastric (poultry and swine) farmers. Data regarding the age class, education, time spent in practice, proficiency in technology, size of the herds, keeping system and level of automation were not collected as participants’ data was kept anonymous. Only gender was considered to ensure equity. During the focus group, participants were asked to complete a SWOT related to the adoption of telemedicine. SWOT acronym for Strengths, Weaknesses, Opportunities and Threats and it originally aims to identify internal and external factors impacting a company’s performance (10). In this study, we adjusted the aim of the SWOT to veterinary practice. Participants were divided in two groups and asked to fill a SWOT matrix with post-it notes, answering the questions: “What are your current strengths and weaknesses to implement veterinary telemedicine, and what potential opportunities and threats do you foresee in its implementation tomorrow?” SWOT analysis aims to identify internal and external resources to implement telemedicine and associated limitations. In our study, participants’ completion of the SWOT matrix allowed to obtain consensus on main ideas. To ensure that the ideas of all participants were heard, the facilitators actively listened to the discussions and prompted the more reserved participants. Additionally, when the report was sent out afterward, participants were informed that they could add any important information they felt was missing.

During this half-day workshop sessions, two other activities were conducted in addition to the SWOT focus groups but not analysed in this study. The description of the workshop’s activities is detailed on Table 1.

**Table 1:** Description of the three activities conducted during the focus groups.

Activities	Activity 1: Current habits		Activity 2: SWOT	Activity 3: Business model	
	Veterinarians	Farmers	Veterinarians and Farmers	Veterinarians	Farmers
Aim	Discuss their current working habits, relation to the client and experience of veterinary telemedicine	Discuss their current working habits, relation to the veterinarian and experience of veterinary telemedicine	Fill a SWOT matrix to answer the question “what are you strengths, weaknesses, opportunities and threats to implement veterinary telemedicine”	Fill a business model canvas with an example of service	Discuss services that telemedicine could enable.
Setting	Group discussion + notes on a paperboard.		2 sub groups with post-it notes and sharing of ideas	2 subgroups with canvas matrix or post-it notes	

Each focus group was intended to last 3.5 h and the entire interview process was recorded. Sheets and post-it notes from each activity were collected at the end of the focus groups. Interviews were conducted in French between October and November 2023. Quotes were then translated into English for this paper, and proofread by a native English speaker to ensure consistency of meaning.

### 2.5 Data analysis

All paper matrices and post-it notes from each activity were digitized into Excel sheets. Recordings were transcribed manually. The data from the SWOT matrices (post-it notes and recordings transcripts) were analyzed using a thematic analysis with both an inductive and a deductive approach. Themes emerged from the focus groups. Then the COM-B model framework (7) was used to classify and refine the theme according to the COM-B categories (psychological capability, physical capability, social opportunity, physical opportunity, reflective motivation, automatic reflection). Each theme was defined in a codebook (Supplementary material). Finally, recordings were listened multiple times to validate the main themes identified and identify emerging themes that were not captured with the notes. Interviews were translated into English for the manuscript (Supplementary material - quotes).

## 3 Results

**Figure 1:**
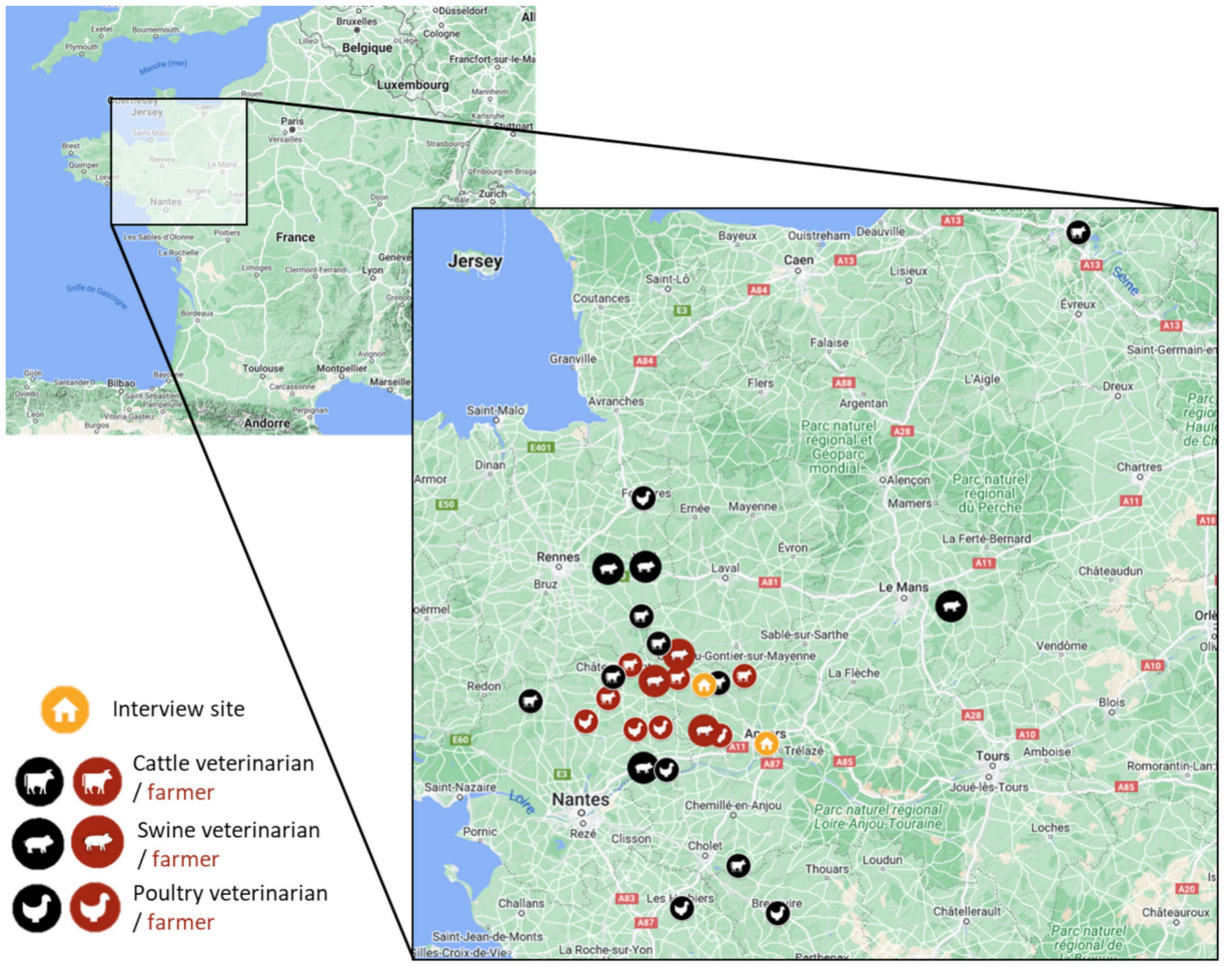
Location of the interview sites and participants’ farm or clinic.

Each focus group consisted of participants with similar professional backgrounds, including veterinarians and farmers, categorized based on species specialization (bovine or monogastric). For the bovine veterinarian focus group, seven veterinarians (two women and five men) were recruited from four French departments (Ille-et-Vilaine, Loire-Atlantique, Maine-et-Loire, and Mayenne). In the monogastric veterinarian focus group, four poultry veterinarians (one woman, three men) and four swine veterinarians (one women, three men) participated from six French departments (Deux-Sèvres, Ille-et-Vilaine, Loire-Atlantique, Maine-et-Loire, Sarthe, and Vendée). The bovine farmers focus group included two dairy cow farmers and two suckler cow farmers (all were men) from two French departments (Loire-Atlantique and Maine-et-Loire). In the monogastric farmers focus group, four poultry farmers (two women and three men) and three swine farmers (all men) were recruited from three French departments (Loire-Atlantique, Maine-et-Loire, and Mayenne). Locations are reported on the following map (Figure 1).

The duration of each focus group averaged approximately 3.5 h, consistent with the targeted timeframe. In the results, only the most explicit quotations have been included in the main text; however, a complete set of quotations for each theme discussed is available in the Supplementary material. One out of four recordings was illegible, necessitating reliance on our notes for analysis. Additionally, the voices in the recordings were indistinguishable.

### 3.1 Physical capability

One theme emerged in relation to physical capability and was related to the development of new skills. This topic was discussed among farmers, who saw telemedicine as a chance to learn new skills from veterinarians. While it was discussed as a minor motivator for farmers to consider adopting veterinary telemedicine, they expressed interest in learning to take immediate action during emergencies. One farmer explained: *“Sometimes you do not have time to wait and you’ll have to act quickly… Having contact* via *video and letting ourself be guided to make the right move allows him [the veterinarian] to see what we are doing.”* Veterinarians also discussed this theme, primarily focusing on regulatory aspects, which will be addressed in section 6 (reflective motivation – regulatory framework).

### 3.2 Psychological capability

Three main themes emerged in relation to physical capability and were related to the veterinarians’ expertise, relation to the farmer, and technologies. The first theme was seen as a facilitator by most veterinarians and farmers. However, opinions were less homogeneous for the second theme; it was seen as a facilitator by some veterinarians and farmers and as a barrier for others. The third theme was mainly seen as a barrier by veterinarians and farmers.

Many veterinarians reported that their expertise developed during their training was a strength for the implementation of telemedicine. They have the scientific knowledge to implement this practice: *“We usually have a data-focused scientific education. We have a background we are not purely clinicians.”*

Moreover, some veterinarians stated that in addition to having expertise, they know their clients. Indeed, having a relationship with farmers could help implementing telemedicine. This familiarity fosters effective communication and adapted support for farmers. One producer explained: *“He knows the farmer as well; I imagine that from one farmer to another, the vet adapts his language, his support.”* However, some veterinarians also explained that even though they have the expertise, unfamiliarity with the farmer (e.g., new client) could pose a significant barrier.

Veterinarians and farmers encounter significant barriers due to difficulties in adopting and familiarizing themselves with new technologies. Veterinarians consider the complexity of existing tools and IT gateways a major weakness, leading to frustration and time loss: *“As soon as it’s complicated and complex to set up, vets do not like it, so it’s a real weakness.”* Similarly, some farmers struggle with technology, which hinders their motivation to adopt veterinary telemedicine effectively. Moreover, the rapid turnover and constant evolution of technology exacerbate these challenges, making it a major barrier for both parties. However, even though adapting to technology could be challenging, some veterinarians explained that the tools are available and easily accessible. One veterinarian explained: *“We already have the tools to do the basic work.”*

Farmers perceive the lack of qualifications among farm employees as a minor barrier to veterinary telemedicine adoption. This concern stems from potential language barriers during communication with veterinarians.

### 3.3 Physical opportunity

Five themes emerged in relation to physical opportunity and were related to the lack of network, the access to tools, the creation of data, difficulty to get paid, the assessment of the intervention and the distance between the veterinary clinic and the farm. The first theme was seen a major barrier by most farmers and veterinarians. The three following themes were mainly discussed between veterinarians as major barriers. The two last themes were seen by both farmers and veterinarians as major facilitators to the implementation of veterinary telemedicine.

Most veterinarians and producers mentioned that telemedicine could allow quicker interventions in remote areas, overcoming the geographical barrier. One farmer explained: *“[technology] can be useful to determine whether to trigger an intervention or not especially if your clinic is 20 kilometres away and not densely populated with farms.”* However, such technology requires the network to be good. Most veterinarians and producers indicated that the network coverage in France was inadequate to practice telemedicine, as the Internet is not available everywhere. A bovine veterinarian emphasized this issue, highlighting the impracticality of teleconsultations due to inconsistent connectivity: *“We do not always have access to 5G or 4G, so in my everyday practice, a teleconsultation is simply not feasible.”*

Moreover, many veterinarians and producers thought that accessibility to tools that could be useful for telemedecine could be difficult. Veterinarians mentioned that there are disparities between producers regarding the ownership of phones, computers and other technologies. One veterinarian explained: *“some farmers still do not have a mobile phone.”* Veterinarians also face challenges due to incompatible software and equipment. Financial constraints further hinder technology adoption, as highlighted by concerns about the high initial investment and ongoing costs. A farmer with 50 dairy cows explained: *“The problem is that actually the base costs (such as internet) remain the same regardless of whether I have a hundred cows or more so as a result the cost per cow can be enormous [for smaller sized farms]. This can complicate the profitability of the venture.”* This complex interplay of factors poses a major barrier to the effective implementation of telemedicine in veterinary practice.

Most veterinarians and producers explained that they have difficulties with the legal aspect of the data generated with telemedicine and to deal with the amount of data that could be generated. Indeed, many veterinarians face challenges in accessing and interpreting data due to legal constraints: *“On data, I spend my time cracking stuff, you know, being in the illegal realm, I use codes that aren’t mine.”* Farmers on the other hand worried about the overwhelming volume of data: *“we do not know which ones to look at anymore,”* and the security of their data: *“if there are image transmissions, they should not be hacked.”*

Three minor facilitators were identified concerning physical opportunity. Veterinarians recognized the potential for data valorization to improve animal health outcomes. Farmers highlighted the opportunity afforded by telemedicine to reduce consultation costs and carbon emissions, a sentiment echoed by veterinarians, underlining a shared interest in the adoption of veterinary telemedicine.

### 3.4 Social opportunity

One main theme emerged regarding social opportunity and was related to a fear of veterinarians to see their profession change. Indeed, they expressed concerns about the potential evolution of their profession, questioning whether future practitioners would be confined to deskwork, disconnected from practical field experience. A bovine veterinarian explained: *“what are we going to end up doing? Will future vets just be guys sitting behind their screens looking at farming data, saying ‘this is not going well, it’s off track’? I find it hard to believe that we could become a profession of non-doers. I’m part of it, you know, I’m behind screens, lots of computers, but at some point you have to keep a connection with doing.”* This raised concerns about the profession’s image and its appeal to young people.

However, some saw these changes as minor facilitators to the implementation of veterinary telemedicine, with a potential attractiveness for different individuals to the profession. Additionally, both farmers and veterinarians saw the adoption of telemedicine as an opportunity to project a modern image of the profession. Farmers believed it could attract young people to farming, given the technological advancements in the field, while veterinarians saw it as a means to showcase a modern clinic and enhance collaboration within the veterinary community through data and case sharing.

### 3.5 Automatic motivation

Two main themes emerged regarding automatic motivation and were related to human contact and levels of adoption of technology. Both themes were identified as barriers to the implementation of veterinary telemedicine. Concerning human contact, both farmers and veterinarians expressed a shared fear that veterinary telemedicine might disrupt the human connection, highlighting concerns about losing the personal interaction inherent in traditional veterinary care. A bovine farmer who emphasized the importance of face-to-face communication with their veterinarian echoed this sentiment: *“I prefer to see [the veterinarian] in person, but if we have no choice, it’s still practical. I like to be in contact with the person and discuss; you delve a little deeper, and he will better understand what needs to be done.”* However, despite these concerns, there is recognition among some participants that telemedicine could potentially strengthen the bond between farmers and veterinarians by complementing current visits rather than replacing them entirely.

Concerning the discussion on the adoption of new technologies, it is evident that proposals inducing significant changes often encounter opposition, a phenomenon commonly referred to as resistance to change (11). Both veterinarians and farmers voiced apprehensions regarding the democratization of technology and differential levels of technological proficiency. A bovine farmer indicated: *“Technology is not yet ubiquitous. Some people are more or less sensitive to it. I like technology, but some I do not even talk to them about it. Some have tried calving sensors and do not want to hear about it, whereas I think it’s great. Everyone approaches technology differently.”* These discussions underscore the heterogeneous nature of technology adoption within the veterinary domain, with some individuals embracing digital tools while others exhibit resistance to change.

### 3.6 Reflective motivation

Five main themes emerged in relation to reflective motivation and were related to regulation of veterinary telemedicine, responsibility, delegation of acts, fear of losing control and risks for animal health. All were considered as major barriers to the implementation of veterinary telemedicine.

The regulatory framework surrounding veterinary telemedicine, particularly regarding the delegation of veterinary acts, stands as a crucial consideration. While certain acts are allowed to be performed by individuals other than veterinarians, unauthorized delegation constitutes illegal practice and raises significant ethical and welfare concerns. Veterinarians express apprehension that the ease of telemedicine may inadvertently facilitate such unauthorized delegation, thereby compromising animal welfare and violating ethical standards. Thus, there is a pressing demand among veterinarians for clear regulations to ensure proper implementation and prevent potential misuse of telemedicine practices: -*"So there needs to be a well-framed delegation of tasks.” -"That’s it, I think the delegation of acts can be a real opportunity but there are still things that are unclear”* Despite recognizing the potential benefits of regulated delegation, such as improved accessibility and efficiency, some veterinarians remain cautious about the potential loss of control over the integrity of veterinary practice.

Moreover, discussions on animal welfare during telemedicine consultations have highlighted concerns regarding medication use and the risk of overlooking underlying farm issues without in-person assessments. A farmer indicated: *“Failure to address the problem as a whole. When there is a problem, such as with a pig or something like that, if no one visits, the veterinarian cannot see if there is a problem with other aspects of the farm in terms of feeding, ventilation, and so on. They may focus on just the autopsy aspect of the problem, but maybe there’s a problem with the farm itself. We treat the immediate problem, but the underlying cause, which may be inherent to something else, is not addressed and it can happen again.”* A notable concern raised by a bovine veterinarian revolves around the responsibility implications of monitoring farming data, raising questions about accountability and potential errors.

Two minor themes were also discussed: the fear of an increased workload for farmers and veterinarians and the potential for enhanced animal health. The first theme was identified as a minor weakness by both farmers and veterinarians. They expressed concern about the potential increase in workload and mental burden associated with telemedicine. A veterinarian highlighted the risk of adding new tasks without considering the impact on work-life balance. Conversely, certain farmers recognized the potential of telemedicine to optimize treatments, leading to improved animal health.

## 4 Discussion

This study aims to investigate the factors influencing the adoption of veterinary telemedicine for the livestock sector in France. To the best of our knowledge, the literature is scarce regarding the perspectives of both veterinarians and farmers on this topic, especially within the context of a regulatory environment where telemedicine is still prohibited. Through qualitative analysis using the COM-B model of behavior change, this study attempted to fill this gap by identifying significant barriers and facilitators to the adoption of veterinary telemedicine.

The qualitative research design using focus groups and the COM-B model provided a robust framework for investigating the barriers and facilitators to veterinary telemedicine adoption. This approach allowed for an in-depth exploration of stakeholders’ perspectives and behaviors, offering insights into the factors influencing telemedicine implementation. However, the qualitative nature of the study limits generalizability, and the COM-B model may not capture all relevant factors. Future research should complement qualitative methods with quantitative approaches. The recruitment strategy prioritized proximity, potentially limiting participant diversity. What’s more, a convenience sampling strategy was used, potentially inducing biases such as the lack of representativeness (12). Unforeseen circumstances caused low attendance in one focus group, potentially biasing results. Additionally, technical issues with one recording necessitated reliance on notes, introducing a potential bias in data collection. Moreover, we could not meet gender equity of participant, as there were 5 female veterinarians for 10 men and two female farmers for nine men. However, in livestock production, there are 1.44 times more male livestock veterinarians than female livestock veterinarians (13) and three times more male producers than females producers (14). Therefore, we were close to the national distribution. Finally, we acknowledge that the opinion of more reserved participants may be less heard, potentially leading to an incomplete understanding of the group’s dynamics and potentially reinforcing dominant views while marginalizing diverse perspectives (15). Despite these challenges, focus group discussions and SWOT analyses allowed for comprehensive exploration of stakeholders’ opinions. Thematic analysis guided by the COM-B model facilitated systematic identification of key themes, though potential researcher bias exists.

While farmers and veterinarians primarily focused on barriers to the adoption of veterinary telemedicine in livestock management during the focus groups, participants also acknowledged some potential benefits. Among the advantages cited were the ability to receive immediate advice in emergency situations, facilitating timely intervention while awaiting the veterinarian’s arrival at the farm. Furthermore, participants acknowledged the potential of telemedicine to assist in determining the necessity of a physical visit from the veterinarian, thereby optimizing resource allocation and minimizing unnecessary travel. In addition, the use of available technology was seen as a means to enhance animal health by facilitating communication, diagnostics, and treatments. These findings resonate with literature, particularly in contexts where farms face a scarcity of qualified veterinarians (16). The alignment between the perspectives of study participants and existing literature underscores the potential benefits of veterinary telemedicine in addressing challenges related to livestock management. Becker et al. (17) shed light on an encouraging trend amid the COVID-19 pandemic: heightened interest and increased technology usage among practitioners for veterinary telemedicine. This shift emphasizes the growing significance of embracing innovative solutions in veterinary care, further supported by industry (18). Therefore, both the participants of the focus groups and existing literature recognize the potential benefits of veterinary telemedicine for enhanced animal health and welfare. However, a few barriers still exist and will be further detailed in the following paragraphs.

One major barrier discussed by veterinarians was the lack of regulation regarding the implementation of telemedicine in the veterinary field. The findings regarding regulation stemming from the focus group discussions shed light on the apprehensions and concerns among veterinarians reflecting concerns about the impact of telemedicine implementation on the veterinary profession and animal health, particularly due to the reduced on-farm examination of animals, which may lead to less precise assessments. To address those concerns, it’s crucial to establish clear regulations. Avignon and Fanuel (19) emphasized this need in France, stressing precise definitions and strict oversight to prevent misuse and maintain ethical standards. For instance, the regulatory framework in North America provides insights into alternative veterinary telemedicine regulation. Indeed, telemedicine is authorized within the Veterinarian-Client-Patient Relationship (VCPR) context, allowing remote consultations and service charges under several conditions. These include the veterinarian’s familiarity with the client and recent working knowledge of the animal, a mutual trust relationship between the client and the veterinarian, and ensuring the animal benefits from appropriate care, medication, and treatment (20). Moreover, insights from Becker et al. (17) underscore the importance of a transparent and well-defined legal framework in facilitating the integration of veterinary telemedicine. The authors highlight the need for clear communication and legal clarification regarding the authorization of digital procedures in veterinary medicine, echoing concerns raised in our study. Their findings suggest that the lack of clarity surrounding the legal framework may act as a constraint on practitioners, hindering the widespread adoption of telemedicine. Addressing these regulatory ambiguities and providing clear guidelines is therefore crucial in fostering greater confidence and participation among veterinarians in utilizing telemedicine. The insights gleaned from both our study and Becker et al. (17) in Germany highlight the pressing need for transparent and well-defined regulatory frameworks to guide the integration of veterinary telemedicine. In France, as in Germany, there exists a shared challenge of navigating regulatory ambiguities and providing clear guidelines to support practitioners in adopting telemedicine practices effectively. Drawing inspiration from the regulatory model observed in North America, particularly the Veterinarian-Client-Patient Relationship (VCPR) context, presents a promising avenue for addressing these concerns. By implementing a similar regulatory approach, France can not only address veterinarian apprehensions and ensure continuity of care but also meet the escalating demand for telemedicine services among farmers.

Another significant barrier to the implementation of veterinary telemedicine is the prevailing attitude toward change among veterinarians and farmers. The focus groups revealed considerable reluctance to adopt new technologies, with concerns ranging from potential technical issues to time constraints and varying rates of adoption. Resistance to change is a common response to proposals for significant changes, often driven by factors like a preference for routine and fear of losing control, even when the proposed change aligns with individuals’ interests (21, 22). In the field of veterinary medicine, despite the existence of this resistance, there is a noticeable demand for telemedicine services, with 51% of farmers expressing interest in getting access to it (23). However, negative attitudes toward telemedicine persist, as highlighted by Avignon and Fanuel (19) in their study, where 84.8% of veterinarians expressed reluctance to experiment with telemedicine, with 36% of them stating that they are against it. Nevertheless, high satisfaction rates among both farmers (99%) and veterinarians (62%) who have experienced telemedicine underscore their interest in continuing to utilize and integrate telemedicine into their regular practice (23). Therefore, facilitating their initial experience with telemedicine is crucial, as it frequently leads to a sustained commitment to its adoption. Implementing a secure regulatory framework can serve as a catalyst in promoting wider participation, fostering trust, and ensuring the successful integration of telemedicine into veterinary practice.

During the focus group sessions, various barriers to the adoption of veterinary telemedicine were identified, including concerns about diminished human contact, delegation of veterinary tasks, fear of losing control, and potential risks to animal health. However, regulatory frameworks, such as those observed in North America, where telemedicine is authorized only within the Veterinarian-Client-Patient Relationship (VCPR), offer promising solutions to these obstacles (20). The Federation of Veterinarians of Europe (FVE) also supports this regulatory approach, advocating for the continuation of physical consultations alongside telemedicine services (24). They stress that telemedicine should be seen as complementary to traditional veterinary visits rather than a replacement. This nuanced perspective may facilitate the acceptance of telemedicine among veterinarians. Therefore, contrary to concerns about reduced human interactions, telemedicine can actually strengthen proximity healthcare and reinforce bonds adding remote contact to physical visits. Moreover, regulatory oversight can address worries related to delegating veterinary tasks, mitigating the risks of deregulation highlighted during the focus groups. By addressing these concerns and ensuring animal welfare, regulatory frameworks can facilitate the successful integration of telemedicine into veterinary practice.

The lack of reliable network connectivity emerged as another significant barrier to the widespread adoption of veterinary telemedicine, as highlighted by both veterinarians and farmers during the focus group discussions. Concerns were voiced regarding the inadequacy of network infrastructure in rural areas, hindering seamless communication and access to telemedicine services. However, studies such as Chastant et al. (23) have indicated that while some farms indeed experience significant network deficiencies (5%), the majority are adequately connected (68%) in France. Furthermore, technological advancements, as explored by Koma et al. (25) offer promising solutions to address connectivity challenges, including innovative approaches to enhance network coverage and reliability, including inside the buildings. Moreover, aside from areas where network coverage is entirely absent, simple solutions can be deployed to circumvent connectivity issues, emphasizing the potential for pragmatic strategies to overcome this barrier to telemedicine adoption.

The final barrier discussed pertains to the access to tools and data, a challenge highlighted by both farmers and veterinarians during the focus group sessions. Farmers expressed concerns about the cost of these tools and the proliferation of software options, while veterinarians echoed these sentiments and added complexities associated with accessing farmer data for surveillance purposes. Chastant et al. (23) revealed a notable gap between farmers’ willingness to share data (63%) and the actual practice, with only 6% regularly sharing data due to concerns about data usage and technical difficulties. However, the study also found that basic technology such as mobile phones sufficed for teleconsultations, with 96% of consultations conducted via this medium during the 2020 experimentation, garnering satisfaction from both farmers and veterinarians. Therefore, an initial step in overcoming this barrier could involve implementing telemedicine through readily available devices like phones and existing applications. Subsequently, further investigation could explore methods to centralize information, streamlining processes for both veterinarians and farmers.

In this study, we have identified key barriers to the adoption of veterinary telemedicine. Moving forward, it is imperative to effectively address these barriers. The Behavior Change Wheel (BCW) provides a structured method for characterizing and designing behavior change interventions (7). Utilizing this framework, along with strategies aimed at shifting attitudes toward telemedicine, will be essential for overcoming resistance and promoting widespread adoption. In doing so, engagement of diverse stakeholders, including veterinary council members, policymakers, and governmental representatives, becomes essential to formulate and implement policies facilitating widespread telemedicine adoption while ensuring regulatory compliance and animal welfare.

## 5 Conclusion

In light of the growing interest in veterinary telemedicine amid technological advancements and limited veterinarian availability, this study explored its adoption factors in France. Identified barriers encompassed technological challenges and regulatory complexities. Conversely, facilitators included veterinarians’ expertise and the potential for quicker interventions. Clear and comprehensive regulations are pivotal for addressing concerns related to veterinary telemedicine in order to provide veterinarians with favorable working conditions and ensure animal welfare.

## Data Availability

The datasets presented in this article are not readily available to preserve the anonymity of the participants. Requests to access the datasets should be directed to AG, ana.guintard@oniris-nantes.fr.

## Footnotes

^1^entitled “Facilitate a focus group to gather the perspectives of a group”: https://idele.fr/detail-formation/formation-animer-un-focus-group-pour-recueillir-le-point-de-vue-dun-groupe
