# Supplementary material for: Barriers and facilitators to implementing veterinary telemedicine in animal production
Source: Front Vet Sci. 2024 Nov 20;11:1452653. doi: 10.3389/fvets.2024.1452653 (PMC11614830; doi:10.3389/fvets.2024.1452653)
Supplement: Supplementary file 1 [file Table_1.DOCX]

**Codebook – Identifying barriers and facilitators to implementing veterinary telemedicine in swine, poultry, and bovine production using a COM-B approach**

| **Capacity to implement veterinary telemedicine** | Refers to capacity as a factor for adopting veterinary telemedicine |
| --- | --- |
| Development of new skills *(physical capability)* | Refers to the farmers' interest in acquiring new skills related to performing acts on animals |
| Knowledge of farms  *(psychological capability)* | Refers to the knowledge that farmers and veterinarians have of the farms, the herds and the scientific knowledge regarding animal health |
| Knowledge of tools *(psychological capability)* | Refers to the knowledge of digital tools and the ability to use them |
| **Opportunity to implement veterinary telemedicine** | Refers to opportunity as a factor for adopting veterinary telemedicine |
| Availability of data *(physical opportunity)* | Refers to the availability of tools to access and valorise health data |
| Value of remote services *(physical opportunity)* | Refers to any type of valorisation of remote services and prices associated |
| Speed of case management *(physical opportunity)* | Refers to all factors influencing the time to provide care or treatments to animals |
| Image of the profession *(social opportunity)* | Refers to any factor influencing the image of farmers and veterinarians, affecting the attractiveness of those professions |
| **Motivation to implement veterinary telemedicine** | Refers to motivation as a factor for adopting veterinary telemedicine |
| Human contact *(automatic motivation)* | Refers to any factor affecting human interactions |
| Attitude towards change *(automatic motivation)* | Refers to all the unconscious factors that drive individuals to change their behaviour |
| Regulatory framework *(reflective motivation)* | Refers to any factor link to regulation and legislation governing veterinary telemedicine |
| Animal welfare *(reflective motivation)* | Refers to all factors that impact animal welfare |
