# Supplementary material for: Barriers and facilitators to implementing veterinary telemedicine in animal production
Source: Front Vet Sci. 2024 Nov 20;11:1452653. doi: 10.3389/fvets.2024.1452653 (PMC11614830; doi:10.3389/fvets.2024.1452653)
Supplement: Supplementary file 2 [file Table_2.DOCX]

| **COM-B category** | **Theme** | **Facilitator/Barriers** | **Major/minor** | **Quote** |
| --- | --- | --- | --- | --- |
| Physical Capability | Development of new skills | Facilitator | Minor | *"Sometimes, you don't have time to wait and you’ll have to act quickly… having contact via video, maybe he would say 'okay, go ahead and do that right away' and letting yourself be guided [...] to make the right move, and he sees what we're doing. Because sometimes on the phone 'what did you do?' 'I don't know, it's bleeding everywhere', we could make the wrong move, but the fact that he has visual [contact] is an advantage."* |
| Psychological capability | Veterinarian expertise | Facilitator | Major | *“We usually have a data-focused scientific education. We have a background we are not purely clinicians. There's the preparatory classes aspect, the higher education establishments (grandes écoles), the versatility of the training."* |
|  | Relation to the farmer | Facilitator and barrier | Major | *"It's true that our vet knows the farm well, knows the animals"*  *"He knows the farmer as well; I imagine that from one farmer to another, the vet adapts his language, his support."*  *"but can't we imagine a base in France, in one place where guys do this while your vet is coming? But it would be complicated to manage, I think, he doesn't know the context."* |
|  | Technologies | Barrier | Major | *"In terms of weaknesses, internally, it's the existing tools, the API communications, it's all about the IT gateways that can be Greek to us and make us lose a lot of time. As soon as it's complicated and complex to set up, vets don't like it, so it's a real weakness."*  *"I include 'farmers' demands and reality' in the weaknesses, because for many guys, they're not there yet, they already struggle to use their phone... for them, it's abstract."*  *"I was reflecting on it, the ventilation in my building, it took me a lot of time to understand how my tool worked. Now I am much more free because I am in control."*  *"We lack training, we're behind"*  *"We may feel overwhelmed, it takes time to acquire the skills to use it."*  *"We have boxes and PCs everywhere on the farm. Except that, in fact, I get into a new thing, like, 'this one works like this...' sigh"*  *"[There is] a rapid turnover, you've barely gotten used to one machine and you're starting again, it evolves very quickly. It's likely to keep changing constantly."*  *"And it has to be easy to use because sometimes it's convoluted... sometimes I think they could have made it simpler, I'm struggling, I spend time on it"* |
|  |  | Facilitator | Minor | *"We already have the tools to do the basic work,"* |
|  | Lack of qualification among farm employees | Barrier | Minor | *"In our farms, we have more and more unqualified employees. Me, on the phone, I will understand the vet, but the employee won't”*  *“I think it could be a barrier for some employees to call the vet, in terms of language for example. Technical language. It [the barrier] probably already exists, but if the employee fears it will turn into a teleconsultation, they will be even more apprehensive, I really feel it*."  *"If it came to telemedicine, I would have a hard time delegating it to someone else other than myself."* |
| Physical opportunity | Lack of network | Barrier | Major | *"We don't always have 5G, we don't always have 4G, so in my everyday practice, a teleconsultation is simply not possible. There's a guy I sometimes can't contact when I'm on the farm and can't find him."*  *"In terms of weaknesses, if you don't have internet coverage, the white zones, the lack of fiber, there are places you know you won't be able to."*  *"In terms of weaknesses: access to 5G, 4G, 3G. Access to the phone,"*  *"Inside the buildings, the pig buildings have concrete walls, it has to go through,"*  *"In the aviaries, it's the same, you have a massive amount of metal that's so significant."* |
|  | Access to tools | Barrier | Major | *"some farmers still don't have a mobile phone."*  *"the lack of technological means because apart from the phone... [...] it's a bit poor,"*  *"more tools but they are not compatible. Even just the business software, we all have a different one, so that's not very annoying, but is the business software compatible with the Lely software? That's a real problem, it goes back to wasting time."*  *"it tires me just to open the thing,"*  *"and it doesn't work on a tablet, it only works on a computer."*  *"The cost of technological equipment."*  *"The problem is that all the cameras, you need to have Wi-Fi in your building, it still costs you a lot, it's also financial."*  *"I'm at the stage where I don't have collars on my cows yet, and I'm thinking about this subject, collars and heat detectors plus ruminal activity and so on, and the problem is that actually the base costs (such as internet) remain the same regardless of whether I have a hundred cows or more as a result the cost per cow can be enormous. And so the profitability of the thing is complicated. I'm looking into whether I should invest money into this or if I should take on another part-time job."* |
|  | Creation of data | Barrier | Major | *"On data, I spend my time cracking stuff, you know, being in the illegal realm, I use codes that aren't mine."*  *"It's not better to have too much data, we don't know which ones to look at anymore,"*  *"with the robot, if you want to exploit everything, you spend half the morning in front of the computer,"*  *"too much data overwhelms the data."*  *"that some people take control of certain things,"*  *"if there are image transmissions, they shouldn't be hacked."* |
|  | Difficulty to get paid | Barrier | Major | *"In terms of weaknesses, I would include the difficulty in getting acceptance, on one hand, and in simply getting paid for consultancy and teleconsultation services. Essentially, it's about getting paid for something other than just travel expenses or medication”*  *“Yes, how much do we charge for that?”*  *“To care in handling your cases, you end up doing a lot of tele-whatever, as an additional service, in follow-up, and in addition to that, either you're not paid or you don't invoice.”*  *“There's also our history, meaning, the current valorization of the work, now, how can we backtrack in a company where we already use telemedicine to varying degrees, where we haven't imagined that it could become a future source of profitability or haven't considered removing it from our current business model to reinvent it? And the weight of history makes it difficult, when you did something for 10-15 years, to make a turn that will be profitable.”*  “*In our internal weaknesses, there's still the organisation and the business model we created, which is nonexistent. How are you going to backtrack tomorrow with clients you had on the phone and you were running your business like that, saying 'excuse me but now we're going to switch to a paid platform at 0.05 cents per minute’. The valuation is a weakness.”* Or *“I am unable to tell a farmer 'from now on, it's paid’."* |
|  | Assessment of the intervention | Facilitator | Major | *"In the strengths, it's the speed of case management, telemedicine allows us to react more quickly. The quality of service, I'll put that in the quickness but also in the teleexpertise."* |
|  | Distance farm-clinic | Facilitator | Major | *“[technology] can be useful to determine whether to trigger an intervention or not especially if your clinic is 20 Kim’s away and not densely populated with farms”*  *"Well, when the vet says he's coming, half an hour later he's in the yard with his boots on, but if he says he's coming and it's in an hour, it's different. For those far away, it can sometimes allow you to start something, even if it doesn't replace the vet's intervention."* |
|  | Data valorization | Facilitator | Minor | *"An increase in performance in collective medicine because precisely, access to data facilitates diagnosis."*  *"As an opportunity, there may also be an increase in the baggage to share with the outside, that is to say, the fact of collecting data allows sharing clinical cases, publications, things to enrich the image of the clinic. There is an image of modernity."*  *"Facilitate common interprofessional reflection, meetings, sharing."* |
|  | Consultation costs | Facilitator | Minor | *“if veterinarians save time on the road, in efficiency and all, there may be some prices that will improve too".*  *"the opportunity would be that it could lower costs a bit. Telemedicine, if there's no travel involved, how is that billed afterwards?"* |
|  | Carbon footprint | Facilitator | Minor | *"We talked a bit about the carbon footprint, so fewer people on the roads if there are no vets coming to the site."* |
| Social opportunity | Profession change | Barrier | Major | *"it's good to be able to progress, we can't be a hindrance to technology, but what are we going to end up doing? Will future vets just be guys sitting behind their screens looking at farming data, saying 'this is not going well, it's off track'? I find it hard to believe that we could become a profession of non-doers. I'm part of it, you know, I'm behind screens, lots of computers, but at some point you have to keep a connection with doing."*  *"there are concerns about the perception of the profession: will the profession not undergo profound changes, and there will be a different attraction, perhaps not necessarily lesser, but different for these practices, and it will discourage some who saw something else in the veterinary profession, so there are psychological barriers."* |
|  | Attractiveness of the profession | Facilitator | Minor | *"Young people nowadays are better than us with technology, it can be an asset... It interests them."*  *"Around machines, technologies have evolved tremendously, and we can see that it still attracts young people. Machines, technology, and all that still attract."*  *"A modern image of the clinic... with data sharing, clinical case sharing within the veterinary community, via the tools available to us and social networks."*  *"Collecting data allows sharing of clinical cases, publications, things to enrich the clinic's image. There's a modern image."*. |
| Automatic motivation | Human contact | Barrier | Major | *"I prefer to see [the veterinarian] in person, but if we have no choice, it's still practical. I like to be in contact with the person and discuss; you delve a little deeper, and he will better understand what needs to be done."* |
|  |  | Facilitator | Minor | *"we see it as a way of practising that strengthens our proximity, that's really the key word."* |
|  | Levels of adoption of technology | Barrier | Major | *"Technology is not yet ubiquitous. Some people are more or less sensitive to it. I like technology, but some I don't even talk to them about it. Some have tried calving sensors and don't want to hear about it, whereas I think it's great. Everyone approaches technology differently."*  *"Not everyone is connected, actually. For some people, it's really complicated." "Both vets and farmers."*  *"We want 100% of vets to be on electronic scheduling, but no, some are still using paper schedules. And that's not right. But we can't get everyone on board."* |
| Reflective motivation | Regulation | Barrier | Major | *"If we have a well-applied regulatory framework, we can imagine well-regulated delegations of veterinary acts in the future. That is to say, the delegation of acts in rural areas, all veterinarians can have diverse and varied positions, but it will be positive for the profession if it is framed for the profession and thought for the profession and not for competitors."*  *"Regulation would address responsibility, but rather as a weakness" "I would say rather as a threat, poor legal regulation and it therefore amounts to misuse."*  *"It's good to have a regulatory framework, but if there is no control, no sanction and if this regulatory framework only concerns a part of the people who will be involved in pseudo-telemedicine, it won't serve much purpose. Subsequently, we could have massive deregulation of the unregulated veterinary act and it won't be positive for the veterinary profession or for farmers in the long run because there will be diagnostic problems."* |
|  | Responsibility | Barrier | Major | *"if you start monitoring their farming data, everything you do, you're somewhat relieving the others of responsibility... it's responsibility, if there's a mistake."*  *"it's difficult in a profession that is so under pressure to see it as an opportunity. We already can't do everything we want."* |
|  | Delegation of acts | Barrier | Major | *"It's both a threat and an opportunity, it depends on how the delegation of tasks is created. The delegation of acts for me it can kill the profession, when I hear that we do remote ultrasounds, it's not the fact of not doing the ultrasounds that bothers me, it's that it's yet another door that will close[...] we will only have emergency cases left and no one will want to do just that, we'll shut down the business and we'll focus on small animal practice."*  *"So there needs to be a well-framed delegation of tasks." "That's it, I think the delegation of tasks can be a real opportunity but there are still things that are unclear."* |
|  | Fear of losing control | Barrier | Major | *"We're all scared, but if we don't do it, we'll lose control, and it will be imposed on us"*  *"the competition that could seize the topic and set its own rules and misuse it."*  *"if we don't seize the opportunity, it will pass us by. And we don't really know how to seize that..."* |
|  | Risks for animal health | Barrier | Major | *"Failure to address the problem as a whole. When there is a problem, such as with a pig or something like that, if no one visits, the veterinarian cannot see if there is a problem with other aspects of the farm in terms of feeding, ventilation, and so on* *They may focus on just the autopsy aspect of the problem, but maybe there's a problem with the farm itself. We treat the immediate problem, but the underlying cause, which may be inherent to something else, is not addressed and it can happen again"* |
|  | Increased workload | Barrier | Minor | *"by always wanting to do more, we can have a problem with mental load, difficulty in assuming all the new tasks that we give ourselves. For generations that want to couple quality work with quality of life. We have to be careful."*  *"we find it practical, we have cameras, except that we are always looking at them, so in the end, we can't escape work."*  *"You always have this mental burden and it's impossible to delegate to a third party, so the increase in digital is an increase in mental load."*  *"how am I going to integrate tools into my daily practice without increasing my workload? I already have enough."*  *"I've been doing this for 15 years and I think that before, in the first years when I started, we only had the phone, maybe a message on the answering machine, and that was all we had to respond to. Now we have farmers sending emails. I have farmers who send me an email in the morning and call me in the evening, 'haven't you seen your emails?' no, I haven't seen my emails." "email, text, WhatsApp, everything!"* |
|  | Enhancing animal health | Facilitator | Minor | *"We often forget that we are food producers, our end customer is the consumer, and if we have means to use fewer antibiotics, fewer products in general, [...] we already use less than before. But telemedicine should not lead us to more systematic treatments; it should lead us to more specific ones. Enhancing quality. Sanitary quality is crucial here."* |
